# Contrasting patterns in the occurrence and biomass centers of gravity among fish and macroinvertebrates in a continental shelf ecosystem

**DOI:** 10.1002/ece3.7150

**Published:** 2021-02-05

**Authors:** Kevin D. Friedland, Szymon Smoliński, Kisei R. Tanaka

**Affiliations:** ^1^ Northeast Fisheries Science Center Narragansett RI USA; ^2^ Demersal Fish Research Group Institute of Marine Research Bergen Norway; ^3^ Department of Fisheries Resources National Marine Fisheries Research Institute Gdynia Poland; ^4^ Pacific Islands Fisheries Science Center National Oceanic and Atmospheric Administration Honolulu HI USA

**Keywords:** center of gravity, habitat, Northeast US Large Marine Ecosystem, random forest, temperature

## Abstract

The distribution of a group of fish and macroinvertebrates (*n* = 52) resident in the US Northeast Shelf large marine ecosystem were characterized with species distribution models (SDM), which in turn were used to estimate occurrence and biomass center of gravity (COG). The SDMs were fit using random forest machine learning and were informed with a range of physical and biological variables. The estimated probability of occurrence and biomass from the models provided the weightings to determine depth, distance to the coast, and along‐shelf distance COG. The COGs of occupancy and biomass habitat tended to be separated by distances averaging 50 km, which approximates half of the minor axis of the subject ecosystem. During the study period (1978–2018), the biomass COG has tended to shift to further offshore positions whereas occupancy habitat has stayed at a regular spacing from the coastline. Both habitat types have shifted their along‐shelf distances, indicating a general movement to higher latitude or to the Northeast for this ecosystem. However, biomass tended to occur at lower latitudes in the spring and higher latitude in the fall in a response to seasonal conditions. Distribution of habitat in relation to depth reveals a divergence in response with occupancy habitat shallowing over time and biomass habitat distributing in progressively deeper water. These results suggest that climate forced change in distribution will differentially affect occurrence and biomass of marine taxa, which will likely affect the organization of ecosystems and the manner in which human populations utilize marine resources.

## Graphical abstract

Habitat models for marine fish and macroinvertebrates were used to estimate occurrence and biomass center of gravity (COG). The COGs of occupancy and biomass habitat were separated by significant distances and trended over time. These results suggest that climate change forced change in distribution will differentially affect occurrence and biomass of marine taxa, which will likely affect the organization of ecosystems and the manner in which human populations utilize marine resources.

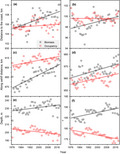

## 1 INTRODUCTION

It is understandable to assume an equivalence between the centers of distribution of where a species occurs versus where biomass is concentrated. However, foundational work in marine ecology would suggest that it might be fortuitous for that to occur. In the development of distribution theory, species distribution has been described as an irregular basin where the extent of the species distribution circumscribed population centers, represented conceptually by shallow and deep portions of the basin (MacCall, 1990). This so‐called “basin model” is based on an irregular basin shape because that is what we encounter in nature. Hence, only if a species' basin was perfectly symmetric would we expect the centers of distribution of occurrence and biomass to align with each other.

Species movement within and between marine ecosystems has taken on both ecological and societal significance (Carozza et al., 2019; Savo et al., 2017). Yet where species have some likelihood of occurring may not define where their most active centers of recruitment are located or where growth may be maximized (Galaiduk et al., 2017; Majoris et al., 2018). The poleward movement of species has generally been described as a thermal accommodation, and with warming conditions, generally more habitat within the thermal constraints of a species becomes available (Wolfe et al., 2020). However, other factors defining habitat may change with these warming trends and fail to provide food resources or competitive advantage for a particular species (Sánchez‐Hernández & Amundsen, 2018). The competitive dynamic in the formation of habitat would likely drive the center of biomass distribution into some subset of the overall occurrence envelope for the species (Majewski et al., 2017).

A changing relationship between the concentration of biomass and the extent of a species distribution would have a number of important ramifications. Against the backdrop of climate change, many species may currently include harvestable concentrations of biomass in accessible habitats; however, as range expands and shifts to new habitats occur, biomass may move to less accessible areas (Smith et al., 2017). For example, depth may define accessibility for a species in the sense that access via a bottom tending gear may be limited by the depth it can be fished, or, become the gear may become too expensive to operate if the target species moves to progressively deeper waters. We have already foreshadowed this potential effect in that the distributional response of species to climate change includes a general movement to higher latitudes (Pinsky et al., 2013), but also has been described to include a movement to deeper depths (Perry et al., 2005). Spatial indicators that can be provided with available analytical techniques are not typically utilized in the management even though they can reduce uncertainties and risks in the management process (Rufino et al., 2018).

Long‐term monitoring survey data can be successfully used for the assessment of the shifts in the distribution of marine fish and macroinvertebrates over time (Thorson & Barnett, 2017). Estimation of the centers of gravity (COGs) is one of the approaches commonly applied in marine ecology (Kendall & Picquelle, 1990; Murawski & Finn, 1988). They have been used to evaluate impacts of climate, fishing pressures, or other anthropogenic factors on the mean location of marine resources (Adams et al., 2018; Perry et al., 2005; Pinsky et al., 2013). However, an extension of that approach has been to use observational data to develop species distribution models to advance inference on the distribution of habitat (Laman et al., 2018). This has taken the form of classification and regression models utilizing a range of mathematical forms that have the ability to capture the dynamics of distribution with inclusion of a range of dynamic predictor variables including physical and biological parameters (Robinson et al., 2017). This approach has been used, for example to effectively describe the effects of temperature on Walleye pollock (*Gadus chalcogrammus*) in the Bering Sea (Thorson et al., 2017).

The US Northeast Shelf continental shelf ecosystem in many ways uniquely provides an opportunity to test hypotheses related to the differentiation of occurrence and biomass centers of distribution. The Northeast US marine ecosystem, which includes some of the most important fishing grounds in the Northwest Atlantic (Sherman & Skjoldal, 2002), has experienced one of the fastest‐warming trends in the world (Pershing et al., 2015). The NES is characterized by a biogeographical transition between subtropical and subpolar biomes and when viewed from a “basin” perspective, the NES is geographically and hydrodynamically complex; hence, it is an irregular basin likely to offer different habitats that match the production requirements of different species (Townsend et al., 2006). The scientific surveys for this system have been comprehensive, which can be used to inform species distribution models (SDMs) for resident species at the microscale (Desprespatanjo et al., 1988). With these factors in mind, this study system should allow us to describe and differentiate centers of occurrence and biomass distribution with sufficient resolution to understand if and how they differ and what sort of change has occurred with centric distributions over time.

Capitalizing on decadal multispecies monitoring efforts, we provide a spatially and temporally comprehensive assessment of the relationship between the occurrence and biomass COGs for a group of fish and macroinvertebrates (*n* = 52) on the US Northeast Shelf. We hypothesized that: (a) the species distribution characterized by occupancy and biomass centers of gravity are not equivalent and that differences between their locations change in time under the changing climatic conditions; and, (b) part of the variation in the fish spatial distribution, and in turn discrepancies between occupancy and biomass COGs, can be explained by the fish size effect. Utilizing a series of machine learning‐based SDMs, three COG metrics (depth, distance to the coast, and along‐shelf distance) were developed to characterize species distributions based on occurrence probability and biomass catch per unit effort weightings. The difference between occurrence and biomass centers were analyzed as in between separation distances. Finally, the relationship between size and depth distribution for these species was analyzed.

## 2 METHODS

### 2.1 Study system

**FIGURE 1:**
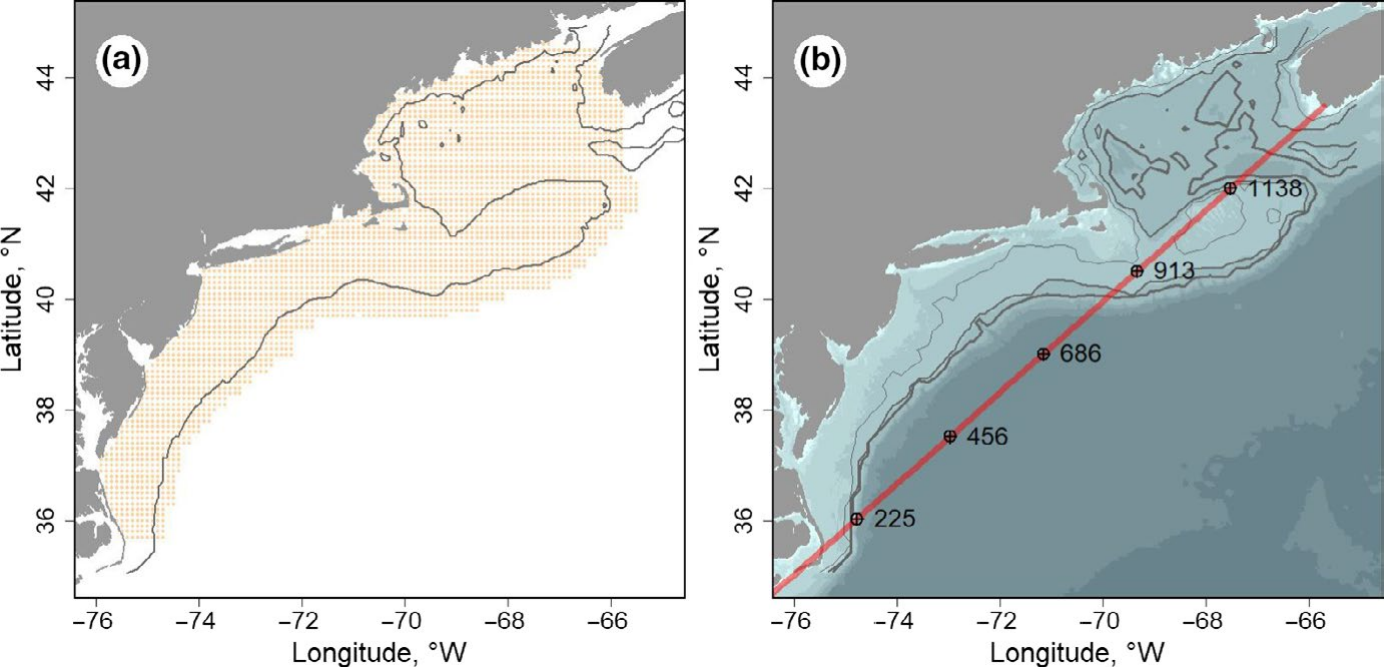
The study system showing the estimation grid (*n* = 3,127) used for occupancy and biomass habitats (a); solid line is 100 m depth contour. Bathymetry of the study system with 50, 100, and 200 m depth contours as thin to thicker lines, respectively (b); red line represents along‐shelf reference line with reference distances (km). Along‐shelf distance reference line represents the distance from the origin of a transect originating at 76.53W 34.60N extending to 65.71W 43.49N. Selected along‐shelf distances are indicated with black dots and corresponding values (in km)

We studied the distribution of fish and macroinvertebrates occurring in the Northeast US Continental Shelf ecosystem (NES: ~278,780 km^2^; 63.33–81.41°W; 28.78–44.87°N), a well‐studied continental shelf marine system along the western boundary of the North Atlantic Ocean. The NES is bounded by the Gulf Stream to the southeast and the U.S. coast to the northwest. These waters are comprised of mixed slope and shelf waters and can be divided into several relatively distinct regional subsystems but are all interconnected to some degree by the Labrador Current, which flows southward toward the equator (Townsend et al., 2006). We analyzed the distribution of fish and macroinvertebrates at spatial scale of 0.1 decimal degree grid (~8–10 km), termed the estimation grid, shown in Figure 1a. Since depth is an important factor, bathymetric relief and key depth contours are shown in Figure 1b. Because the NES is oriented on a diagonal in respect to latitude and longitude, simply using latitude would seem insufficient to describe poleward movement. Following Nye et al. (2009) we used along‐shelf distance as a metric to describe progress along this SW to NE corridor. The reference line used to determine along‐shelf distance is shown in Figure 1b.

### 2.2 Distributional response variables

This study is based on a series of SDMs for taxa captured in a multi‐decade fishery‐independent bottom trawl survey conducted in the NES. The biannual bottom trawl survey has been conducted by the Northeast Fisheries Science Center each year since 1963 in the fall (September–November) and in the spring (March–May/June) since 1968, occupying upwards of 300 stations during each season and is based on a random stratified design (Desprespatanjo et al., 1988). Catches were standardized for various correction factors related to vessels and gears used in the time series (Miller et al., 2010). The survey catch provided the binary response of absence or presence for each taxa as the response variable in occurrence models and catch per unit (log_10_(CPUE kg tow^−1^ + 1)) as a continuous variable in the regression models estimating biomass habitat (see Data Availability Statement). We applied log‐transformation to the biomass data, because nonconstant variation of a response variable may give greater weight to data with higher variation during random forest model training (De'ath & Fabricius, 2000). Despite the availability of survey catch data back to the 1960s, the time series of data used in model fitting and to estimate habitat distributions was limited to the period 1976–2018, which as described below, is limited by the length of predictor variable time series. Prior to the analysis, the survey data were screened to exclude surveys lacking key information (e.g., geographic coordinates, tow duration, water temperature). This preanalysis data screening process resulted in survey data containing over 36,000 tows.

### 2.3 Predictor variables

Physical and biological environmental data used as predictor variables included dynamic variables that changed annually with recurring sampling and static variables that were held constant over the model‐training period. The suite of predictors can be summarized over five general categories listed in Table 1.

**TABLE 1:** Summary of predictor variables used in the development of spring and fall occupancy and biomass habitat models

Predictor variable categories	Description	Number
Physical environment variables	Physical and oceanographic variables including depth, surface and bottom temperature, and surface and bottom salinity derived from surveys	5
Benthic terrain descriptors	A series of variables that characterize the structure of benthic habitats, most of which are based on bathymetry data. See Table 2 for details	19
Secondary production variables	Abundance of zooplankton taxa and a zooplankton biomass index (settled bio‐volume) composed mostly of copepod species. Some taxa only identified to family or others to a general category. See Table 3 for details	19
Remote sensing Primary production variables	Remote sensed measurements of monthly mean chlorophyll concentration; and, the gradient magnitude or frontal data for the same fields	24
Remote sensing Physical environment variables	Remote sensed measurements of monthly mean SST; and, the gradient magnitude or frontal data for the same fields	24

Note

Number refers to number of variables.

Physical environmental variables including surface and bottom water temperature (°C) and salinity (PSU) were made contemporaneously with survey trawl samples with Conductivity/Temperature/Depth (CTD) instruments (see Data Availability Statement). Temperature and salinity were initially tested as dynamics variables; however, salinity was found to be a weak predictor (Friedland et al., 2020) and was applied as a static variable, which enabled training and fitting the models over the time period 1976–2018. Depth of the survey station (meters) was treated as a static variable in the analysis.

Benthic terrain descriptors included a series of static variables that characterize the shape and complexity of the substrate. Most benthic terrain variables were derived from the depth measurements, such as vector ruggedness, rugosity, and slope (Table 2). Other variables described the substrate itself, such as benthic sediment grain size. The vorticity of benthic currents was also considered a benthic terrain variable.

**TABLE 2:** Summary of benthic terrain predictor variables used in the development of spring and fall occupancy and biomass habitat models

Variable	Notes	References
Complexity—Terrain Ruggedness Index	The difference in elevation values from a center cell and the eight cells immediately surrounding it. Each of the difference values are squared to make them all positive and averaged. The index is the square root of this average	Riley et al. (1999)
Namera bpi	BPI is a second order derivative of the surface depth using the TNC Northwest Atlantic Marine Ecoregional Assessment (“NAMERA”) data with an inner radius = 5 and outer radius = 50	Lundblad et al. (2006)
Namera_vrm	Vector Ruggedness Measure (VRM) measures terrain ruggedness as the variation in three‐dimensional orientation of grid cells within a neighborhood based the TNC Northwest Atlantic Marine Ecoregional Assessment (“NAMERA”) data	Hobson (1972)
Prcurv—2 km, 10 km, and 20 km	Benthic profile curvature at 2 km, 10 km and 20 km spatial scales was derived from depth data	Winship et al. (2018)
Rugosity	A measure of small‐scale variations of amplitude in the height of a surface, the ratio of the real to the geometric surface area	Friedman et al. (2012)
seabedforms	Seabed topography as measured by a combination of seabed position and slope	http://www.northeastoceandata.org/
Slp—2 km, 10 km, and 20 km	Benthic slope at 2 km, 10 km and 20 km spatial scales	Winship et al. (2018)
Slpslp—2 km, 10 km, and 20 km	Benthic slope of slope at 2 km, 10 km and 20 km spatial scales	Winship et al. (2018)
soft_sed	Soft‐sediments is based on grain size distribution from the USGS usSeabed: Atlantic coast offshore surficial sediment data	http://www.northeastoceandata.org/
Vort—fall (fa), spring (sp), summer (su), and winter (wi)	Benthic current vorticity at a 1/6 degree (approx. 19 km) spatial scale	Kinlan et al. (2016)

In addition to the dynamic station temperature variables, remote sensing sea surface temperature (SST) fields were used to derive a complimentary set of static physical environment variables. SST fields from the MODIS Terra sensor were used to generate monthly mean SST data and monthly gradient magnitude, or frontal fields of the SST (see Data Availability Statement). There are many methods used to identify fronts (Belkin & O'Reilly, 2009) in oceanographic data that usually utilize some focal filter to reduce noise and then identify gradient magnitude with a Sobel filter. Calculations were performed in R using the ‘raster’ package (version 2.6‐7) by applying a three by three mean focal filter and a Sobel filter to generate x and y derivatives, which were then used to calculate gradient magnitude.

Biological covariates included predictor variables representing lower trophic level primary and secondary production. Primary production variables were monthly chlorophyll concentration static variables developed from remote sensing data sources. The chlorophyll concentration data included measurements made with the Sea‐viewing Wide Field of View Sensor (SeaWiFS), Moderate Resolution Imaging Spectroradiometer on the Aqua satellite (MODIS), Medium Resolution Imaging Spectrometer (MERIS), and Visible and Infrared Imaging/Radiometer Suite (VIIRS) sensors during the period 1997–2016. These data were merged using the Garver, Siegel, Maritorena Model (GSM) algorithm (Maritorena et al., 2010) obtained from the Hermes GlobColour website (see Data Availability Statement). As with the remote sensing SST data, monthly gradient magnitude (i.e., frontal chlorophyll fields) were also developed.

Secondary production covariates were based on zooplankton abundances measured by the Ecosystem Monitoring Program, which conducts shelf‐wide bimonthly surveys of the Northeast U.S. Shelf ecosystem (Kane, 2007). Zooplankton are collected obliquely through the water column to a maximum depth of 200 m using paired 61‐cm Bongo samplers equipped with 333‐micron mesh nets (see Data Availability Statement). Sample location in this survey is based on a randomized strata design, with strata defined by bathymetry and along‐shelf location. Plankton taxa are sorted and identified to the lowest possible taxonomic rank. We used the density estimates (number per 100 m^3^) of the 18 most abundant taxonomic categories and a biomass indicator (settled bio‐volume) as potential predictor variables (Table 3). The zooplankton time series has some missing values, which were ameliorated by summing data over five‐year time steps for each seasonal period and interpolating a complete field using ordinary kriging. Thus, for example, the data for spring 2000 would include the available data from tows made during the period 1998–2002.

**TABLE 3:** Summary of zooplankton predictor variables used in the development of spring and fall occupancy and biomass habitat models

Variable name	Full name
acarspp	*Acartia* spp.
calfin	*Calanus finmarchicus*
chaeto	Chaetognatha
cham	*Centropages hamatus*
cirr	Cirripedia
ctyp	*Centropages typicus*
echino	Echinodermata
evadnespp	*Evadne* spp.
gas	Gastropoda
hyper	Hyperiidea
larvaceans	Appendicularians
mlucens	*Metridia lucens*
oithspp	*Oithona* spp.
para	*Paracalanus parvus*
penilia	*Penilia* spp.
pseudo	*Pseudocalanus* spp.
salps	Salpa
tlong	*Temora longicornis*
volume	Plankton bio‐volume

### 2.4 Occupancy and biomass habitat models

Species distribution models were developed using essentially the same approach as reported in (Friedland et al., 2020), with the only differences being the application of salinity variables as static fields instead of dynamic ones and the length of the training data time series. SDMs were fit using random forest machine learning (Cutler et al., 2007), which were implemented using the *‘randomForest’* R package (version 4.6‐14; Liaw & Wiener, 2002). Random forest models have been demonstrated to achieve a greater predictive power compared to other statistical models (Smolinski & Radtke, 2017). Prior to fitting the model, the independent variable set was tested for multi‐collinearity among the predictors, and variables were eliminated using *'rfUtilities'* R package (version 2.1‐5; (Evans & Cushman, 2009)). From this reduced set of predictors, the final model variables were selected utilizing the model selection criteria of Murphy et al. (2010) as implemented in rfUtilities. The occupancy models were evaluated for fit based on out‐of‐bag classification accuracy using the Area Under the ROC Curve (AUC) index using the *'irr'* R package(version 0.84.1; (Gamer et al., 2019)) using the default classification threshold probability of 0.5. Biomass model regressions were evaluated for fit using the root mean squared error statistic based on the R package ‘*Metrics*’ (version 0.1.4; (Hamner et al., 2018)). The candidate species were limited to the consistently abundant taxa from the survey, which was defined as those species occurring in at least 150 trawl tows and which numbered 96 species. From this candidate list, a subgroup of species deemed to have occurrence models with satisfactory fits were used to estimate occupancy and biomass habitats over the estimation grid for the same period of the training data, 1976–2018.

### 2.5 Distribution indices

Change in distribution was characterized with three spatial distribution metrics derived from to occurrence probability and biomass distribution for modeled taxa. The three COG metrics: distance to the coastline (DTC), along‐shelf distance (ASD) and depth of occurrence (DEPTH) were calculated based on the predictions of the random forest models. Model‐based estimates of COGs have been shown to be more robust than metrics calculated on the raw data (Thorson et al., 2016). COGs can be calculated as:

$${\text{COG}}_{\text{year}}=\frac{\sum_{i=1}^{k}\left(d_{i}\times t_{i}\right)}{\sum_{i=1}^{k}t_{i}}$$

where *d_i_* is a value of DTC, ASD or DEPTH associated with an estimation grid *i*. *t_i_* denotes either modeled occurrence or biomass at estimation grid *i*. *k* is the total number of estimation grid cells in the study area (*n* = 3,127).

The distribution metrics of COG were calculated as the weighted mean distances or depths using the occurrence probability or biomass measures of the subject taxon as the weighting factor. DTC is the distance of the COG from the closest position on the coastline, expressed in units of km. ASD was taken as the distance from the origin of a transect to the COG position of the subject taxon projected to the nearest point of the transect. The transect originates at 76.53°W 34.60°N and extends to 65.71°W 43.49°N (Figure 1b). For this index, lower ASD correspond to positions in the southwest portion of the ecosystem and higher values more in the northeast. DEPTH represents the depth (depth of the seabed) of the COG and is expressed in units of meters.

Differences between occurrence and biomass COGs were tested for main factor effects using a two‐way repeated measures ANOVA test; factors included model type (biomass, occupancy), year, and species. Trend in each distribution measure was estimated using the generalized least squares model selection approach described in Hardison et al. (2019). This approach fits trend models with Gaussian, AR(1), and AR(2) correlation structures prior to selection by small‐sample AIC (Sugiura, 1978), and reduces estimation bias due to autocorrelated residuals when compared to linear regression or the Mann‐Kendall test alone. In addition to analyzing the distribution metrics, the distance between occurrence and biomass COGs were calculated; these calculations were done on the DTC, ASD and DEPTH indices as well as the COG latitude/longitude positions. Differences between seasonal COGs were tested for main factor effects using a two‐way repeated measures ANOVA test; factors included season, year, and species. Trends in these data were tested in the same manner as the COG measures.

### 2.6 Fish size at depth

The relationship between fish body size and depth of occurrence was calculated for the suite of species modeled. For specimens captured in the bottom trawl survey that included a length measurement (cm), linear models were estimated between length and depth as the independent variable. Regression fits with model *p* < .01 were considered significant.

## 3 RESULTS

### 3.1 Model performance and species selection

A set of 52 species were identified that provided acceptable model fits in both spring and fall seasons and thus formed the basis of a balanced analysis of change and contrast in occurrence and biomass COGs (Table 4). Occurrence model fits had average AUC scores of 0.85 and 0.86 in the spring and fall, respectively. Our intention was to only use species models with an AUC > 0.75; we made one exception and included the species model for Jonah crabs that had a AUC = 0.74 in both seasons. Of all the species used, six taxa, American lobster, Atlantic rock crab, Jonah crab, longfin squid, northern shortfin squid, and sea scallop, were macroinvertebrates whereas the balance were finfish. Though inclusion in the study was based on the performance of the occurrence models, the biomass models had relatively high performing fits as well. The average RMSE scores for the spring and fall biomass models were 0.11 and 0.12, respectively.

**TABLE 4:** Taxa modelled for both occurrence and biomass habitat models

Species	Abbr	Spring		Fall		Species	Abbr	Spring		Fall	
		AUC	RMSE	AUC	RMSE			AUC	RMSE	AUC	RMSE
*Sebastes fasciatus*	ACARED	0.93	0.15	0.94	0.14	*Lophius americanus*	MONKFH	0.76	0.16	0.77	0.18
*Alosa pseudoharengus*	ALEWIF	0.75	0.15	0.87	0.10	*Prionotus carolinus*	NORSEA	0.85	0.11	0.85	0.11
*Hippoglossoides platessoides*	AMEPLA	0.91	0.10	0.94	0.10	*Macrozoarces americanus*	OCPOUT	0.79	0.15	0.82	0.08
*Homarus americanus*	AMLOBS	0.79	0.15	0.77	0.17	*Merluccius albidus*	OFFHAK	0.91	0.05	0.93	0.04
*Gadus morhua*	ATLCOD	0.84	0.22	0.89	0.18	*Pollachius virens*	POLLOC	0.82	0.19	0.86	0.17
*Clupea harengus*	ATLHER	0.76	0.21	0.90	0.14	*Cancer irroratus*	RCKCRA	0.80	0.06	0.76	0.04
*Scomber scombrus*	ATLMAC	0.77	0.19	0.77	0.10	*Urophycis chuss*	REDHAK	0.80	0.15	0.82	0.16
*Dipturus laevis*	BARSKA	0.89	0.09	0.88	0.11	*Leucoraja garmani*	ROSSKA	0.93	0.04	0.93	0.04
*Anchoa mitchilli*	BAYANC	0.94	0.04	0.92	0.11	*Stenotomus chrysops*	SCUPZZ	0.90	0.10	0.91	0.16
*Centropristis striata*	BLABAS	0.86	0.07	0.86	0.07	*Hemitripterus americanus*	SEARAV	0.80	0.14	0.81	0.11
*Helicolenus dactylopterus*	BLAROS	0.90	0.06	0.90	0.06	*Placopecten magellanicus*	SEASCA	0.84	0.11	0.84	0.13
*Alosa aestivalis*	BLUHER	0.76	0.09	0.88	0.05	*Chlorophthalmus agassizi*	SHORTP	0.90	0.01	0.93	0.01
*Peprilus triacanthus*	BUTTER	0.86	0.12	0.77	0.23	*Illex illecebrosus*	SHTSQD	0.88	0.06	0.81	0.16
*Scyliorhinus retifer*	CHADOG	0.95	0.04	0.94	0.04	*Merluccius bilinearis*	SILHAK	0.81	0.17	0.82	0.17
*Raja eglanteria*	CLESKA	0.92	0.07	0.92	0.09	*Mustelus canis*	SMODOG	0.92	0.09	0.89	0.17
*Tautogolabrus adspersus*	CUNNER	0.84	0.05	0.87	0.05	*Malacoraja senta*	SMOSKA	0.89	0.07	0.88	0.07
*Lepophidium profundorum*	FAWMEL	0.89	0.04	0.89	0.04	*Squalus acanthias*	SPIDOG	0.79	0.32	0.80	0.28
*Paralichthys oblongus*	FOUFLO	0.87	0.11	0.83	0.13	*Urophycis regia*	SPOHAK	0.88	0.09	0.84	0.13
*Enchelyopus cimbrius*	FRBERO	0.89	0.03	0.88	0.02	*Paralichthys dentatus*	SUMFLO	0.84	0.11	0.90	0.13
*Citharichthys arctifrons*	GULFLO	0.87	0.02	0.86	0.03	*Amblyraja radiata*	THOSKA	0.88	0.12	0.89	0.14
*Melanogrammus aeglefinus*	HADDOC	0.85	0.20	0.84	0.21	*Urophycis tenuis*	WHIHAK	0.87	0.13	0.88	0.14
*Cancer borealis*	JONCRA	0.74	0.05	0.74	0.05	*Scophthalmus aquosus*	WINDOW	0.83	0.12	0.85	0.13
*Urophycis chesteri*	LGFINH	0.92	0.02	0.92	0.02	*Pseudopleuronectes americanus*	WINFLO	0.88	0.13	0.87	0.14
*Leucoraja erinacea*	LITSKA	0.83	0.23	0.86	0.19	*Leucoraja ocellata*	WINSKA	0.81	0.23	0.88	0.17
*Myoxocephalus octodecemspinosus*	LONSCU	0.89	0.13	0.88	0.13	*Glyptocephalus cynoglossus*	WITFLO	0.84	0.09	0.90	0.09
*Loligo pealeii*	LONSQD	0.89	0.12	0.85	0.22	*Limanda ferruginea*	YELFLO	0.87	0.12	0.88	0.12

Note

ABBR is the six‐character code for investigated taxa. Random forest performance statistics for occupancy (Area Under the ROC Curve; AUC) and biomass (Root Mean Square Error, RMSE) model, by species and season.

### 3.2 Change in occurrence and biomass center of gravity measures

**FIGURE 2:**
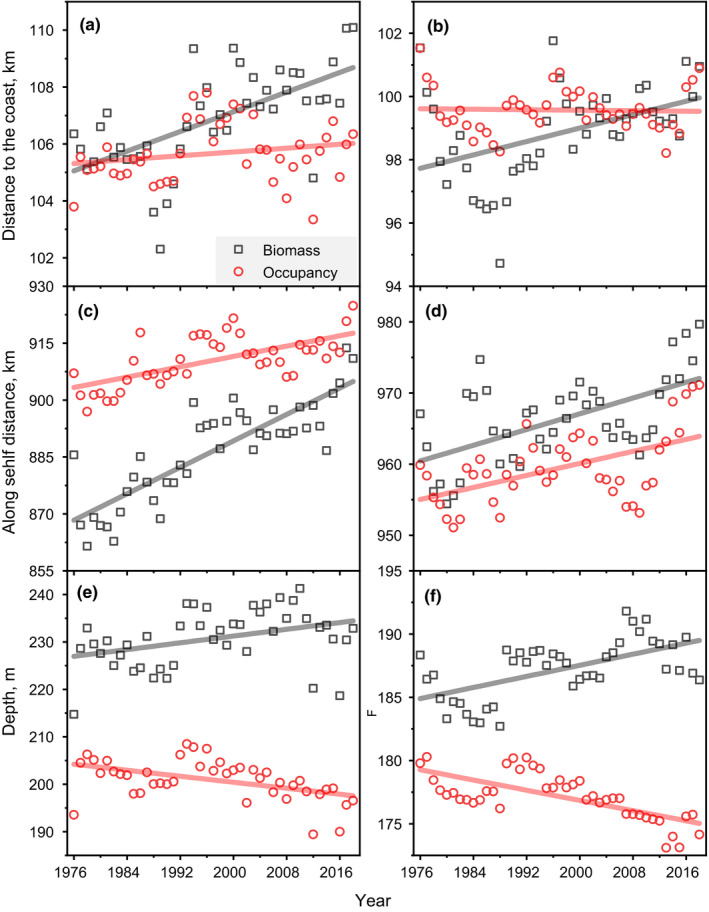
Time series (1976–2018) and linear trends of; mean distance to the coast (DTC) center of gravity index in spring (a) and fall (b); mean along‐shelf distance (ASD) center of gravity index in spring (c) and fall (d); mean depth (DEPTH) center of gravity index in spring (e) and fall (f). Black squares—based on biomass habitat model, red circles—based on occupancy habitat model

The mean COG metrics for occurrence and biomass habitats across species varied seasonally and changed both synchronously and asynchronously. Time series of the mean occupancy DTC metrics were without trend in both spring and fall which was in contrast to increasing trends found in the biomass habitat data (Figure 2a,b). Spring occupancy DTC remained at approximately 105 km throughout the time series and was generally <100 km in the fall. Mean biomass DTC increased in both seasons, but the only significant trend among these times series was found in the spring biomass DTC data (Table 5). The main factor comparison test showed no difference between the occupancy and biomass DTC data in either season (Table 6).

**TABLE 5:** Linear trend in the center of gravity distribution metrics (a) and linear trend in the differences in the distribution metrics between models for occurrence and biomass habitats (b)

Season	Metric	Model	Slope	CI	*p*
(a) Trend in distribution					
Spring	DTC	Biomass	0.087	(0.034, 0.139)	.010
		Occupancy	0.022	(−0.017, 0.060)	.264
	ASD	Biomass	0.838	(0.561, 1.114)	.002
		Occupancy	0.357	(0.134, 0.580)	.014
	Depth	Biomass	0.197	(0.006, 0.388)	.053
		Occupancy	−0.141	(−0.27, −0.012)	.070
Fall	DTC	Biomass	0.032	(−0.046, 0.109)	.469
		Occupancy	−0.006	(−0.049, 0.037)	.770
	ASD	Biomass	0.282	(0.047, 0.517)	.048
		Occupancy	0.229	(0.002, 0.455)	.082
	Depth	Biomass	0.074	(−0.018, 0.166)	.217
		Occupancy	−0.109	(−0.173, −0.046)	.010
(b) Trend in difference					
Spring	Lat/Lon		−0.156	(−0.268, −0.045)	.023
	DTC		0.060	(0.005, 0.115)	.099
	ASD		0.530	(0.430, 0.630)	.000
	Depth		0.340	(0.212, 0.469)	.001
Fall	Lat/Lon		0.023	(−0.084, 0.130)	.669
	DTC		0.043	(0.000, 0.085)	.123
	ASD		0.057	(−0.079, 0.193)	.416
	Depth		0.183	(0.107, 0.259)	.013

Note

Slope with 95% confidence intervals (CI) and associated probability of the slope estimate (*p*) are given by season, metric, and model.

Abbreviations: ASD, along‐shelf distance; DEPTH, the depth of the center of gravity; DTC, distance to the coastline.

**TABLE 6:** Results for main factor comparison of model types using a two‐way repeated measures ANOVA test

Season	Metric	*F*	*p*
Spring	DTC	0.709	.404
	ASD	4.579	.037
	DEPTH	9.668	.003
Fall	DTC	0.297	.588
	ASD	1.059	.308
	DEPTH	3.803	.057

Note

Factors include model type (biomass, occupancy), year, and species; each model had 1, 42, 51 degrees of freedom by factor for a total of 4,471.

Time series of the mean occupancy and biomass ASD metrics all trended to higher or more northeasterly locations in both spring and fall (Figure 2c,d). In spring, the occupancy COGs tended to be more to the northeast than the biomass COGs, while in spring, the positions reversed with the occupancy COGs further to the southwest. ASD trends for both habitats and seasons were significant at *p* ≤ .082 (Table 5). However, the main factor test suggests that the only spring occupancy and biomass ASD metric values were significantly different (Table 6).

Time series of the mean occupancy and biomass DEPTH metrics tended to diverge in both spring and fall, indicating a shallowing of occurrence and a movement of biomass distributions to deeper depths (Figure 2e,f). With the exception of the fall biomass data, all of these trends were significant at *p* ≤ .07, further supporting the observation of divergence in depth distribution (Table 5). The main factor comparison tests confirm the implied differences in occupancy and biomass DEPTH data in both seasons (Table 6). In summary, while occupancy habitat has tended to remain at constant distance from the coast, biomass habitat has moved further offshore. This is also reflected in changes in depth distribution of the habitat types, with biomass habitat tending to distribute in deeper water. The COG for both habitat types have shifted along the NES shelf further to the northeast.

### 3.3 Change in distance between occurrence and biomass centers of gravity

**FIGURE 3:**
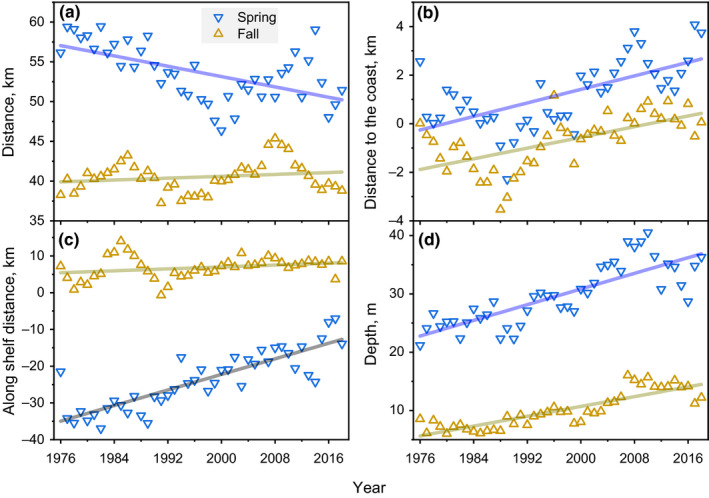
Temporal variations (1976–2018) in mean distance between biomass and habitat centers of gravity latitudes and longitudes (a), difference between distance to the coast (b), along‐shelf distance (c), and depth (d) indices for spring and fall models

The spacing between occupancy and biomass habitat COGs were substantial and, in some cases, changed over time. The distance between occupancy and biomass COGs simply based on their latitude and longitude positions suggest center spacing of approximately 55–40 km in spring and fall, respectively (Figure 3a). The negative trend in the spring spacing distances was significant whereas the fall spacing distance time series was nonsignificant (Table 5). The main factor comparison tests suggest that the spacing in spring and fall were not significantly different (Table 7).

**TABLE 7:** Results for main factor comparison of seasons using a two‐way repeated measures ANOVA test

Metric	*F*	*p*
Lat/Lon	2.658	.109
DTC	1.805	.185
ASD	10.800	.002
Depth	5.670	.021

Note

Factors include season, year, and species; each model had 1, 42, 51 degrees of freedom by factor for a total of 4,471.

The distance between occupancy and biomass DTC COGs indicate the relative position between the COGs in respect to the shoreline. Spring difference in DTC distance begin around zero and increased more than 2 km indicating that the biomass COGs relocated further offshore by that amount (Figure 3b). The fall distances start in negative numbers indicating biomass was further inshore at the beginning of the time series and have now equalized. The distance trend in the spring spacing was significant at *p* = .1 and the fall trend was nonsignificant (Table 5). The main factor comparison test suggests that the spacing in spring and fall were not significantly different (Table 7).

The difference between occupancy and biomass ASD seasonal COGs varied by sign and magnitude. Spring differences in ASD COGs were all negative reflecting the more southeasterly position of biomass COG (Figure 3c). The fall distances were all positive reflecting the reversal of occupancy and biomass relative position; the fall differences were of much lower magnitude of <10 km. The distance trend in the spring spacing was significant at *p* = .01 and the fall trend was nonsignificant (Table 5). The main factor comparison test suggests that the spacing in spring and fall were significantly different (Table 7).

The difference between occupancy and biomass DEPTH seasonal COGs were both positive indicating biomass COGs were distributed at deeper depths. Spring differences in DEPTH were larger than fall and increased from approximately 20 m to nearly 40 m over the time series (Figure 3c). The fall depth differences were all positive but only ranged from approximately 7 to 15 m. The trend in depth difference was significant at *p* < .014 in both seasons (Table 5). The main factor comparison test suggests that the spacing in spring and fall were significantly different (Table 7). To summarize, the overall spacing of occupancy and biomass COGs ranged from approximately 40 to 55 km across seasons; however, no single distribution metric was able to explain this distance in total. Instead, the separation of occupancy and biomass COGs was the result of the combined effects of complex movements in respect to both the along and offshore axes of the ecosystem.

### 3.4 Fish size at depth accounts for differences in centers of gravity

**FIGURE 4:**
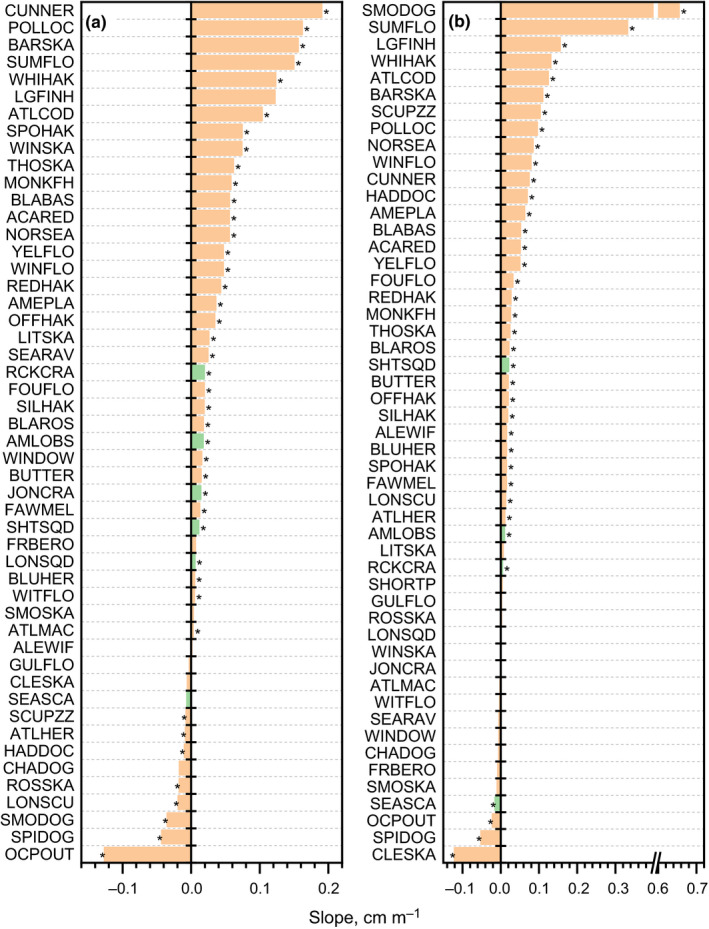
Linear regression slope coefficients between length and depth for fish (beige) and macroinvertebrates (green) for spring (a) and fall (b) seasonal surveys. Species abbreviations are specified in Table 1. Regression with *p* < .01 statistical significance marked with an asterisk

The majority of species were found to have positive relationships between size and depth indicating an ontogenetic distributional gradient. In spring, 50 of the 52 study species had sufficient data to estimate the relationship between size and depth; of the regressions, 38 (76%) had positive slope coefficients (Figure 4a). Slightly more pronounced were the differences in sign of the fall coefficients where 40 (78%) were positive based on 51 taxa with sufficient data (Figure 4b).

## 4 DISCUSSION

The occurrence and biomass centers of distribution were distinct among fish and macroinvertebrates in the US Northeast Shelf large marine ecosystem. These centers tend to be separated by substantial distances and have been shifting dynamically in response to changing climate conditions within the ecosystem. Generally, the two centers were separated by an average distance of around 50 km, which approximates the dimensions of the minor axis of the ecosystem. Clearly, habitat gradients for these species are irregular and cause habitats to be partitioned. Habitat partitioning is most visibly associated with life stage and can be represented with fish size as a proxy variable. Such relationships between body length and habitat have previously been demonstrated for multiple reef fish species with varying life history characteristics (Galaiduk et al., 2017). Due to the limited range of depth (10–40 m), ontogenetic environmental niche partitioning in that study was driven mainly by the physical descriptors of habitat complexity (i.e., local relief and/or slope) and selected biotic variables (i.e., probability of occurrence of mixed undifferentiated vegetation, sessile invertebrates, and reef). However, in our study, depth was one of the most important predictors. Since the depth of fish occurrence was strongly related to fish size, it suggests that fish size indirectly affects the spatial distribution of these marine resources.

Shifts in the location of the COGs for occurrence and biomass among fish and macroinvertebrates presented in this study show contrasting patterns. Discrepancies in the occupancy and biomass can be mediated by the competition for resources, which in turn drives migration of organisms between different areas (Bijleveld et al., 2018). Range of other ecological phenomena can modulate species occupancy and biomass distribution, including abrupt spatial discontinuities or temporal changes in environmental conditions (Brown, 1984) or population dynamics (Foggo et al., 2007). As previously presented for macrozoobenthos in Dutch Wadden Sea, there is no general relationship between species' biomass and occupancy, therefore predicting biomass from its occupancy is not always possible (Bijleveld et al., 2018). Since both features of the species distribution can provide valuable information into the management process but show inconsistent patterns, our results underscore the high value of long‐term programs monitoring both occupancy and biomass of organisms.

Habitat centers of distribution are shifting to higher latitude, which mirrors the shifts seen in empirical distributions; however, partitioning the analysis by occurrence and biomass reveals significant seasonal differences in where biomass is concentrated. The juxtaposition of biomass to occurrence reverses from spring to fall, which reflects the large seasonal excursion of biomass COGs versus occurrence COGs. Biomass tends to shift at distances approaching 100 km, whereas occurrence shifts less than half that amount. There are a number of factors that form latitudinal gradients that could shape the productivity of fish. An obvious gradient is the one formed thermally as the ecosystem cools and warms seasonally.

For some species, changes in the distributional COG may be contrary to what is seen in the main trends. In this ecosystem, most species experienced a shift in distribution to high latitudes or further to the northwest along shore; howevere, for some species like winter skate (*Leucoraja ocellata*) and shortnose greeneye (*Chlorophthalmus agassizi*), trends in ASD COG were negative, indicating shifts to the lower latitudes. For both species, finding deeper water meant movement to the south of their former habitats. Hake species have had COG distributions off the shelf break around 72–70°W; however, now these species have centers of distribution in more inshore areas around 42–43°N (Nye et al., 2009). Consequently, their occurrence COG is more inshore where they impose significant predation pressure on protected species (Friedland et al., 2012). These results suggest that particular species or groups of species may show contrasting patterns in the shifts of their COGs and since multiple ecosystem elements are shifting their distribution at different speeds and directions, it may potentially increase the rate of changes in interspecific interactions within the ecosystem (Kordas et al., 2011).

Environmental factors dominate in the modeling of species distribution and have overshadowed other effects, such as biotic interactions, and specific biological characteristics, like phenotypic plasticity or locomotor performance (Twiname et al., 2020; Zhang et al., 2018). Besides the environmental factors in the SDM framework, we investigated also possible relationships between fish body size and depth of occurrence. Most species were found to have positive relationships between size and depth indicating an ontogenetic distributional gradient. Therefore, part of the variation in the fish spatial distribution, and in turn discrepancies between occupancy and biomass COGs, can be explained by the fish size effect. The differences in occurrence and biomass habitat distributions reflect a response to changing physical conditions in the ecosystem, but also to the underlying ontogenetic organization of populations. The difference in depth distribution of the two habitat types is likely related to the change in depth preference by age and size of most species captured in the survey. Current methodological advancement allows, for example, for the inclusion of additional biological information, and provides hybrid approaches placed between purely correlative and mechanistic models (Bush et al., 2016). Therefore, we encourage further studies, which can incorporate size properties of the studied populations or species (e.g., asymptotic length derived from the von Bertalanffy growth functions commonly used in fisheries science) in the prediction of species redistribution patterns and speed of these processes. Further parallel prediction of the changes in the size structure of the populations (Tu et al., 2018) and inclusion of these predictions in the models of species occupancy or biomass shifts in the future can help to improve their reliability.

The shifts or temporal trends in the COGs and differences between occupancy and biomass COGs were more pronounced in the spring than in the fall. We can attribute these differences to two aspects of the physical environment. Though the spring and fall surveys, and thus the time frames used to inform the models, were spaced by approximately six months or a half year, the surveys occur at different dynamic locations in the annual temperature cycle. The spring survey time frame revolves around April, which tends to be more proximate to the winter minimum than the fall survey time frame in October and its proximity to the summer maximum. As a consequence, the spring habitats are defined on a thermal field with less change, whereas the fall thermal field is changing rapidly. The change in the fall would be cooling, which would likely have the effect of compressing habitats within the range of the ecosystem. Though more commonly described in respect to oxygen stress (Campbell & Rice, 2014), thermal habitat compression has been described (Brown et al., 2016) and may be relevant here. Additionally, there are physical demarcations to the latitudinal extent of the ecosystem that are not artificially imposed by the survey itself. The northern boundary of the ecosystem is associated with the shelf break, thus in the summer months, northerly annual migrations meet depth boundary conditions that may also serve to compress the fall distributions.

The differential shifts in species occupancy and biomass may have ramifications for the spatial management of the ecosystem. On face value, greater concentration of biomass at depth presents a climate‐induced stressor on fishery operations through likely changes in transit times and operating costs (Kleisner et al., 2017). Higher concentrations of biomass at deeper depth will most likely be located further offshore, which is substantiated by the observed trends in biomass and distance to the coast. At the same time, species occurrence footprints have enlarged (Friedland et al., 2020) and occupancy COGs have migrated inshore and into shallower water, thus creating new, accessible fishing opportunities and conflicts with existing management. Temporal shifts and discrepancies of occupancy and biomass COGs in the transboundary areas may cause challenges for the effective management of fisheries and allocations of fishing quotas (Baudron et al., 2020). Unequal distribution of occupancy and biomass habitats of fish and macroinvertebrates can also lead to the unbalanced fishing pressures and mortality. These results suggest that climate change forced change in distribution will differentially affect occurrence and biomass of marine taxa, which will likely affect the organization of ecosystems and the manner in which human populations utilize marine resources.

## CONFLICTS OF INTEREST

The authors declare that there are not conflicts of interest.

## AUTHOR CONTRIBUTION

**Kevin D. Friedland:** Conceptualization (lead); Writing‐original draft (lead); Writing‐review & editing (lead). **Szymon Smoliński:** Conceptualization (supporting); Writing‐review & editing (supporting). **Kisei R. Tanaka:** Conceptualization (supporting); Writing‐review & editing (supporting).

## Data Availability

The bottom trawl survey data, including the size at depth data, are publicly available at NMFS InPort data site (https://inport.nmfs.noaa.gov/inport/). Remote sensing sea surface temperature data is available at NASA Ocean Color site (http://oceancolor.gsfc.nasa.gov/cms/). Remote sensing chlorophyll concentration data is available at the Hermes GlobColour website (hermes.acri.fr/index.php). Zooplankton abundance data is available through the National Centers for Environmental Information website (https://accession.nodc.noaa.gov/0187513).
